# Underrated *Staphylococcus* species and their role in antimicrobial resistance spreading

**DOI:** 10.1590/1678-4685-GMB-2019-0065

**Published:** 2020-02-10

**Authors:** Ciro César Rossi, Monalessa Fábia Pereira, Marcia Giambiagi-deMarval

**Affiliations:** 1 Universidade Federal do Rio de Janeiro, Instituto de Microbiologia Paulo de Góes, Laboratório de Microbiologia Molecular, Rio de Janeiro, RJ, Brazil.; 2 Universidade Federal do Espírito Santo, Departamento de Patologia, Vitória, ES, Brazil.

**Keywords:** Horizontal gene transfer, antimicrobial resistance, coagulase negative staphylococcus, biofilm, CRISPR, One Health

## Abstract

The increasing threat of antimicrobial resistance has shed light on the interconnection between humans, animals, the environment, and their roles in the exchange and spreading of resistance genes. In this review, we present evidences that show that *Staphylococcus* species, usually referred to as harmless or opportunistic pathogens, represent a threat to human and animal health for acting as reservoirs of antimicrobial resistance genes. The capacity of genetic exchange between isolates of different sources and species of the *Staphylococcus* genus is discussed with emphasis on mobile genetic elements, the contribution of biofilm formation, and evidences obtained either experimentally or through genome analyses. We also discuss the involvement of CRISPR-Cas systems in the limitation of horizontal gene transfer and its suitability as a molecular clock to describe the history of genetic exchange between staphylococci.

## Introduction

Every year, hundreds of thousands of deaths around the world are attributed to the ever increasing problem of antimicrobial resistance (Laxminarayan *et al.*, 2016). It is estimated that, if the issue is not properly addressed, by the year of 2050, more than 10 million annual deaths will be caused by antimicrobial-resistant microorganisms, surpassing deaths by cancer (O’Neill, 2014). Although the accuracy of this frightening estimate is questioned by some authors, since the future scenario of disease treatment may be considerably different from the current one, the clinical, economical, and public health burden associated with antimicrobial resistance is undeniable (Kraker *et al.*, 2016; Robinson *et al.*, 2016).

In the context of resistance dissemination, bacteria of the *Staphylococcus* genus, residents of the normal microbiota of human beings and animals, play a central role. *Staphylococcus aureus* is the main pathogen of the group, responsible for a variety of clinical infections in humans and a leading cause of bacteremia, endocarditis, and many infections related to invasive medical devices (Tong *et al.*, 2015). Meanwhile, coagulase negative staphylococci (CoNS), especially *S. epidermidis* and *S. haemolyticus*, have emerged as recurrent causative agents of nosocomial infections, mainly those related to indwelling devices (Becker *et al.*, 2014). They are a serious threat in the twilight of the multidrug resistance era, for actively participating in the horizontal transmission of resistance (Becker *et al.*, 2014; Czekaj *et al.*, 2015).

Genomic analyses indicate that many of the genetic determinants of resistance may have been exchanged between several staphylococcal species from different environments and hosts (Rolo *et al.*, 2017; Rossi *et al.*, 2017a; Kohler *et al.*, 2018;). This includes species that are understudied and/or underestimated, either for having been recently discovered, rarely involved with infectious diseases, or for lacking canonical staphylococcal virulence factors. However, recent evidence shows that these underrated species, despite not being usual pathogens, may have an important role in the exchange of antimicrobial resistance genes, by acting as gene-reservoirs for more pathogenic species, such as *S. aureus* (Otto, 2013; Rossi *et al.*, 2017a, 2019).

Given the increasing recognition of the importance of previously overlooked *Staphylococcus* species, the goal of this review was to present evidences that put these bacteria in the front row of resistance dissemination and highlight their potential threat to human and animal health.

### Antimicrobial resistance increase and the interconnectedness of its spreading

In the past decade, consumption of antibiotic drugs increased by 35%, with 76% of this growth concentrated in Brazil, Russia, India, China, and South Africa (Van Boeckel *et al.*, 2014), with a projection of a rise in consumption in the next 15 years of 67% (Van Boeckel *et al.*, 2015). The disturbing situation of antimicrobial resistance led the World Health Organization to elaborate the “Global action plan on antimicrobial resistance”, with goals that include: (i) improving the understanding of the problem through communication, education and training, (ii) increasing surveillance and research, (iii) advancing in preventive actions to reduce the incidence of infections, and (iv) optimizing the use of antimicrobial drugs in human and veterinary health (WHO, 2017). Tied to this worldwide concern to restrain the dispersion of resistance, the emerging engagement of scientists and other professionals with the “One Health” agenda increases the acknowledgement of the need for global approaches as the only possible way to keep the predicted disaster of 2050 of more than 10 million annual deaths caused by resistant microorganisms from actually happening.

Considered as a “new professional imperative”, One Health is a collaborative and multidisciplinary effort to achieve optimal health for people, animals and our environment from local to global scales (King *et al.*, 2008). It recognizes that the welfare of these three domains is interconnected and this link, ignored for so long, is crucial for the spreading of antimicrobial resistance. The use of antimicrobial drugs in agriculture, for example, is the largest worldwide, thus being a major driver of resistance for several reasons, like exposure of bacteria to sub-therapeutic doses of the antibiotics and the exposure of human and animals to those drugs and microorganisms, either via consumption of products or environmental release (Silbergeld *et al.*, 2008). Studies also point out that the use of antimicrobial drugs, particularly to maintain health, productivity, and promoting growth of food animals, contribute to the dispersion of resistant bacteria in livestock and human beings (Marshall and Levy, 2011; Van Boeckel *et al.*, 2015). In aquaculture, it is estimated that around 80% of antimicrobials used reach the environment with their activity intact, thereby expanding the surroundings where selection of resistant microorganisms will take place (Cabello *et al.*, 2013). Even insects commonly associated with food animals, like houseflies and cockroaches, are presumably vehicles of microorganisms from the farms to urban centers (and vice versa), as evidenced by multidrug-resistant clonal lineages carried by them, that were also isolated from different environments (Zurek and Ghosh, 2014).

Pet animals have been shown to act as reservoirs of resistant bacteria, which in turn act as reservoirs of mobile genetic elements that carry antimicrobial resistance genes (Guardabassi *et al.*, 2004; Rossi *et al.*, 2017a). In fact, the relationship between the animals and their owners significantly shapes the microbiota of both counterparts (Song *et al.*, 2013). For that reason, the indiscriminate use of antimicrobials in veterinary practice represents a direct threat to human beings. More aggravating is the fact that some drugs that are either not recommended to be used in humans, or those that are considered as last resources, are heavily used to treat animals. Some examples include the polymixins and chloramphenicol and its derivatives, the latter presenting several adverse effects that limit its employment (Cabello *et al.*, 2013; Poirel *et al.*, 2017).

Consistently, multidrug resistant strains, like methicillin-resistant *Staphylococcus aureus* (MRSA) are ubiquitous, being isolated from humans, pets, food, other animals and the environment (Vanderhaeghen *et al.*, 2010; Kock *et al.*, 2013; Rossi *et al.*, 2017b). Due to local variations in control practices and specific characteristics of circulating clones, the overall geographic distribution of MRSA, for example, can range from 1 to 5% of isolates in northeastern Europe to more than 50% in certain Latin American countries (Brazil, Uruguay, Venezuela, Bolivia, Peru, and Chile) and in Japan (Lee *et al.*, 2018).

### Variety and clinical significance of *Staphylococcus* spp.

Although *S. aureus* is the major bacterium of its genus, more than 50 *Staphylococcus* species are registered in the *List of Prokaryotic Names with Standing in Nomenclature* database, available at http://www.bacterio.net (Parte, 2018). However, DNA sequencing of complete genomes or housekeeping genes, phylogenetic analyses, DNA–DNA hybridization, protein profiles, and genotyping techniques have constantly led to reclassifications or proposals of new species and subspecies (Sasaki *et al.*, 2007; Fitzgerald, 2009; Taponen *et al.*, 2012). As these techniques advance and new sources of *Staphylococcus* are explored, especially in different animals, new species are also discovered, such as *S*. *nepalensis*, isolated from goats (Spergser *et al.*, 2003), *S. stepanovicii*, isolated from wild small animals (Hauschild *et al.*, 2010), *S. pseudintermedius*, isolated from several domestic animals, such as dogs, cats, horses and parrots (Devriese *et al.*, 2005), and *S. agnetis*, isolated from bovines with subclinical and clinical mastitis (Taponen *et al.*, 2012).

In general, staphylococci are natural inhabitants of skin and mucous membranes of human beings and animals, while the prevalence of species widely varies according to the host. *S. felis*, for example, is typically isolated from feline, either healthy or presenting signs of lower urinary tract disease, otitis externa, and ocular disease (Rossi *et al.*, 2017b; Worthing *et al.*, 2018); *S. pseudintermedius* is prevalent in domestic dogs, healthy or related to diseases like pyoderma and otitis externa (Ruscher *et al.*, 2009; Rossi *et al.*, 2018); *S. caprae* is involved with intramammary infections in dairy goats (Moroni *et al.*, 2005), among others. Regardless of their source, infections caused by unusual human pathogens are sporadically reported (Morfin-Otero *et al.*, 2012; Novakova *et al.*, 2006a,b), with special emphasis on those caused by *S. pseudintermedius*, with most cases indicating the contact of domestic dogs with their owners as the probable source of infection (Van Hoovels *et al.*, 2006; Riegel *et al.*, 2011; Lozano *et al.*, 2017). Given their great adaptability to unfavorable conditions (Uribe-Alvarez *et al.*, 2016; Rossi *et al.*, 2017c), staphylococci isolated from the surrounding environment are also responsible for human acquisition of relevant pathogens, including MRSA (Hardy *et al.*, 2006; Sexton *et al.*, 2006).

The production of coagulase and its plasma-clotting activity is a central diagnostic feature to distinguish staphylococcal strains in coagulase-positive staphylococci (CoPS) and coagulase-negative staphylococci (CoNS) (Becker *et al.*, 2014). In addition to being key to diagnostics and group differentiation of *Staphylococcus* in CoPS and CoNS, coagulase is a virulence factor that leads to the cleavage of soluble fibrinogen to produce a fibrin coat in the surface of the bacteria, thus protecting it from phagocytosis and other host defenses (Powers and Wardenburg, 2014). Moreover, the polymorphisms of the coagulase-coding gene, *coa*, allows it to be explored in molecular typing techniques (Salehzadeh *et al.*, 2016; Javid *et al.*, 2018). However, because some populations of CoPS may not have the *coa* gene, while some CoNS present this gene, the applications of these methods are limited (Almeida *et al.*, 2018). These coagulase-variable strains are more frequently found in some species than others, but their misdiagnosis may lead to unsuitable treatment of infections and control measures. This is especially significant when the detection of the pathogenic *S. aureus* relies on coagulase production and strains of these species do not produce coagulase, leading to isolate misidentification (Sundareshan *et al.*, 2017). Strains of the CoNS *S. chromogenes*, *S. xylosus*, *S. cohnii* and *S. agnetis* have been reported to clot plasma, leading to misidentification of the pathogens causing mastitis in dairy animals (Taponen *et al.*, 2012; Santos *et al.*, 2016; Almeida *et al.*, 2018). For that reason, researchers have relied on more accurate identification methods, including the sequencing of the 16S rRNA and the housekeeping genes *rpoB*, encoding the β subunit of RNA polymerase, as well as *tuf*, encoding the EF-tu elongation factor (Ghebremedhin *et al.*, 2008; Li *et al.*, 2012). Given its simplicity to perform and its cost effectiveness, matrix assisted laser desorption ionization-time of flight mass spectrometry (MALDI-TOF) is emerging as a potential tool for microbial identification and diagnosis (Singhal *et al.*, 2015). MALDI-TOF identification of staphylococci shows a good correlation with sequencing results, although the lack of standards for uncommon and recently identified species is still a bottleneck (Rossi *et al.*, 2017b).

CoPS, with a special emphasis on *S. aureus*, responsible for several clinical infections, from those in skin and soft tissues to systemic disease processes like sepsis (Tong *et al.*, 2015), are considered to be more pathogenic than CoNS. A plethora of virulence factors have been described and extensively reviewed for *S. aureus*, comprising molecules involved in tissue adhesion, immune evasion and host cell injury (Powers and Wardenburg, 2014). Proteins covalently anchored to the cell wall peptidoglycan may participate in not only biotic and abiotic surface adhesion, but also in biofilm formation and iron acquisition, among other functions (Foster *et al.*, 2014). Moreover, a wide variety of toxins can be secreted, aiming to evade the defense mechanisms of the host (Otto, 2014). Other relevant pathogenic CoPS belong in the *Staphylococcus indermedius* group (SIG). This group includes zoonotic pathogens typically associated with dog bites, i.e., *S. intermedius, S. pseudintermedius,* the recently described *S. cornubiensis* (Murray *et al.*, 2018), and *S. delphini*, first isolated from skin lesions of dolphins (Varaldo *et al.*, 1988).

The increasing recognition of the importance of *S. pseudintermedius* as a zoonotic pathogen has boosted investigations on virulence factors involving infections caused by this bacterium. Among them, pore-forming toxins seem to play a pivotal role in the characteristic skin infections (Abouelkhair *et al.*, 2018; Maali *et al.*, 2018). Because many of these virulence factors are encoded in mobile genetic elements (MGE) with extensive variation in gene content, different strains strongly vary in their virulence arsenal (Otto, 2014; Moon *et al.*, 2015).

The CoNS constitute the vast majority of staphylococci, comprising more than 80% of the species described to date (Becker *et al.*, 2014; Parte, 2018). Historically considered as harmless inhabitants of the human and animal microbiota, in the past two decades CoNS have emerged as the major nosocomial pathogens, mostly associated with invasive medical devices (Becker *et al.*, 2014), being particularly threatening to immunocrompromised individuals (Morfin-Otero *et al.*, 2012). Among these opportunistic pathogens, *S. epidermidis* is the most frequent cause of nosocomial infections (Gomes *et al.*, 2014), followed by *S. haemolyticus* (Czekaj *et al.*, 2015). The infections caused by these species are particularly important because they are difficult to treat, since device colonization is usually related to biofilm formation, which can lead to complications, including sepsis, endocarditis, and a wide variety of local infections derived from the bloodstream spreading of bacteria (Chang *et al.*, 2018; Oliveira *et al.*, 2018).

### Commensal and opportunistic staphylococci acting as gene reservoirs

*Staphylococcus* species that are usually considered as harmless inhabitants of the microbiota of animals and humans beings, like most CoNS, lack nearly all of the virulence factors described for *S. aureus* and do not encode many known specific factors, apart from a limited amount of toxins and exoenzymes (Zhang *et al.*, 2003). Their emergent threat comes from the fact that these bacteria may carry a huge amount of antimicrobial resistance genes located in MGEs (Gomes *et al.*, 2014; Czekaj *et al.*, 2015; Hosseinkhani *et al.*, 2018).

Multidrug-resistant CoNS have been increasingly linked to infections outbreaks in healthcare units (Chang *et al.*, 2018; Li *et al.*, 2018), including strains that are not only isolated from patients, but also from healthcare workers, and the environment (Widerstrom *et al.*, 2016). Microbiome studies reveal clonal staphylococcal strains wide ability to colonize diverse hosts and surrounding environments (Song *et al.*, 2013; Lax *et al.*, 2014). Their widespreadness can lead to infections caused by multidrug resistant strains in both humans and animals by species that are atypical pathogens in one of the hosts. Dutta *et al.* (2018), for example, have recently isolated *Staphylococcus pettenkoferi* strains causing peritonitis from a domestic cat. This species was discovered in 2002 in various human patients showing multiple clinical manifestations, but had never been isolated from animals before. Likewise, as aforementioned, the typical canine staphylococcal species *S. pseudintermedius* can occasionally cause disease in human beings as well (Riegel *et al.*, 2011; Lozano *et al.*, 2017).

This long overlooked exchange of microorganisms and antimicrobial resistance between different hosts and environments is now clear, and strains isolated from humans and domestic animals carry several resistance genes in common (Schwarz *et al.*, 2018). Even though some antimicrobials have their use restricted to treat infections in animals, many multidrug resistance genes in staphylococci isolated from them confer resistance to antimicrobial agents that are highly important in human medicine (Wendlandt *et al.*, 2015). Then, even if one staphylococcal species is not a common pathogen, it can be a potential threat, because it may be capable of transferring antimicrobial resistance genes to more pathogenic species, such as *S. aureus*, thereby enhancing its capacity to resist drug therapy (Haaber *et al.*, 2017). For that reason, some CoNS have been suggested to act as antimicrobial genes reservoirs within the *Staphylococcus* genus (Cafini *et al.*, 2016; Rossi *et al.*, 2017a).

The acquisition of antimicrobial resistance genes is mainly credited to the occurrence of conjugation and bacteriophage transduction and the presence of dozens of insertion sequences in staphylococcal genomes, whose rearrangements contribute to genome plasticity and strains’ phenotypic diversification (Takeuchi *et al.*, 2005; Haaber *et al.*, 2017). For a while, bacteriophage transduction was perceived as the primary route of horizontal gene transfer between staphylococci, while conjugation was believed to play only a minor role in the evolution of this genus, given the scarcity of mobilization and conjugation loci in staphylococcal plasmids (Ramsay *et al.*, 2016; Haaber *et al.*, 2017). In fact, only 5% of sequenced staphylococcal plasmids harbor genes required for autonomous transfer by conjugation, which is contrasting with the abundant evidences for horizontal transfer of plasmids between different lineages and species of *Staphylococcus* (Ramsay *et al.*, 2016).

However, new mechanisms of transference of those types of plasmids have been discovered and probably explain the extensive plasmid transfer within the genus. These mechanisms include conjugation mediated by integrative and conjugative elements (ICEs), also referred to as conjugative transposons (Lee *et al.*, 2012), *in trans* recognition of multiple variants of the canonic origin of transfer (*oriT*) by some conjugative plasmids (O’Brien *et al.*, 2015b), and the activity of novel conjugative plasmids described in community isolates of *S. aureus* that are capable of mobilizing unrelated non-conjugative plasmids (O’Brien *et al.*, 2015a).

The Staphylococcal Cassette Chromosome *mec* (SCC*mec*), a genomic island that encodes resistance to methicillin and nearly all other beta-lactam antibiotics, is also a protagonist in the emergence of resistant strains. Analyses of numerous SCC*mec* sequences indicate that this mobile genetic element evolved by recombination and assembly events involving an ancestral SCC*mec* III cassette between strains of the *S. sciuri* group and the species *S. vitulinus* and *S. fleurettii*, which were then transferred to *S. aureus* and other species (Rolo *et al.*, 2017), with CoNS acting as their central reservoirs (Saber *et al.*, 2017).

Our group has demonstrated the transference of high molecular weight plasmids carrying the *mupA* gene for mupirocin resistance from *S. epidermidis, S. aureus,* and *S. haemolyticus* strains, from either human or canine origin, to another *S. aureus* strain (Bastos *et al.*, 1999; Rossi *et al.*, 2016, 2018). Mupirocin resistance spreading is alarming, since this drug, which is used as an intranasal ointment by healthcare workers, can significantly reduce the occurrence of nosocomial infections caused by MRSA (Patel *et al.*, 2009). Similarly, Cafini *et al.* (2016) demonstrated the transfer of linezolid resistance mediated by the *cfr* gene through plasmids, between *S. epidermidis*, *S. aureus,* and *Enterococcus* spp. isolates from Japan. *Enterococcus* spp. is also a protagonist of nosocomial infections and has been pointed out to participate in plasmid exchange with MRSA, through which resistance to the last-resource antibiotic vancomycin appears to be acquired (Kohler *et al.*, 2018). A study performed by Meric *et al.* (2015) with hundreds of genomes of *S. aureus* and *S. epidermidis* showed that these species share only about half of their pan genome, but there was a considerable sharing of mobile genetic elements between the two species, in particular genes associated with pathogenic islands and the SCC*mec.*

Biofilm formation, a characteristic feature of many CoNS (Barros *et al.*, 2015; Buttner *et al.*, 2015), seems to provide the ideal environment for the occurrence of horizontal gene transfer (Madsen *et al.*, 2012).

### Biofilms as the perfect place for horizontal gene transfer among staphylococci

Bacterial biofilms are dense surface-associated cellular communities embedded in a protective self-produced matrix of exopolysaccharides, whose development confers new properties to its inhabitants (Black and Costerton, 2010, Flemming *et al.*, 2016). Biofilms feature a social cooperation between bacteria that enhances nutrient uptake and distribution and, given its protective matrix, hinders the exposure to antimicrobials and host defenses, thus increasing survival (Flemming *et al.*, 2016). Hence, biofilm formation plays a fundamental role in virulence.

Although the biofilm mechanisms involved in resistance to host defenses are not entirely understood, they include spatially limiting the access of leukocytes and their products to the target cells, suppression of leukocyte effector functions and cell-cell communication to increase resistance (Leid, 2009). Reducing membrane permeability also contributes to limit the entrance of antimicrobials (Liu *et al.*, 2000). However, the presence of antibiotics that affect the *Staphylococcus* cell wall have been shown to modulate natural transformation in a process that seems to be dependent of the alternative sigma fator H, SigH (Thi *et al.*, 2016). In fact, the expression of SigH-controlled genes makes *S. aureus* cells competent for transformation by plasmids or chromosomal DNA (Morikawa *et al.*, 2012), which in turn increases the probability of plasmid exchange between the cells within the biofilm. Liu *et al.* (2017) demonstrated that the presence of many antibiotics induces the expression of the *ccrC1* gene, involved with the excision of the SCC*mec* from the bacterial chromosome, thus triggering its transfer.

Overall, the high cellular density of biofilms, increased concentration of exogenous DNA, enhanced cell competence, stabilization of cell-cell contact by the matrix and even the biofilm architecture, that facilitates the dispersion of MGEs, may contribute to horizontal gene transfer (Madsen *et al.*, 2012; Savage *et al.*, 2013). The fact that more transconjugants are produced after filter-matings, when compared with planktonic cells mating, indicates the importance of biofilms for the transfer of MGEs (Madsen *et al.*, 2012). It has been shown that *S. aureus* biofilms can drastically increase the rates of conjugation and transformation of plasmids containing resistance to multiple drugs (Savage *et al.*, 2013).

As observed for many bacteria, during biofilm maturation *S. aureus* can suffer lysis and release its genomic DNA, producing the so-called external DNA (eDNA), which is a major component of the matrix of biofilms established by this bacterium (Sugimoto *et al.*, 2018). The eDNA adsorbs to the surface of a single cell in long loop structures, which act as an adhesive substance that facilitates cell attachment, in addition to influence the hydrophobicity of the bacterial cell surface (Okshevsky and Meyer, 2015). Furthermore, in *S. epidermidis*, eDNA production reduces the depth of vancomycin penetration, as the matrix-embed and negatively charged DNA interacts with positive charges of the antimicrobials (Doroshenko *et al.*, 2014). Moreover, the eDNA released by the cells to constitute the biofilm matrix is also available for horizontal gene transfer by transformation to competent cells in the community (Hannan *et al.*, 2010; Vorkapic *et al.*, 2016).

The dispersion of MGEs and eDNA is likely facilitated by the empty spaces within biofilms, which also function as channels that allow the flowing of fluids and the consequent dispersion of nutrients and oxygen to all cells. Studies indicate that this biofilm architecture is influenced by the production of biosurfactant compounds by the adherent bacteria (Davey *et al.*, 2003). Consistent with this, we have recently shown that *S. haemolyticus* strains are capable of producing biosurfactants that affect biofilm formation on abiotic surfaces (Rossi *et al.*, 2016). Likewise, surfactant peptides produced by *S. epidermidis* control biofilm maturation and detachment from colonized catheters (Wang *et al.*, 2011).

While, as abovementioned, CoNS lack most of the *S. aureus* virulence factors, biofilm formation is a major feature of *S. epidermidis* and *S. haemolyticus,* as well as of other CoNS, such as *S. saprophyticus, S. hominis* and *S. cohnii*. This phenotype is positively correlated with widespread antimicrobial resistance (Allori *et al.*, 2006; Czekaj *et al.*, 2015).

### The CRISPR paradox in gene-reservoirs’ staphylococci

**Figure 1:**
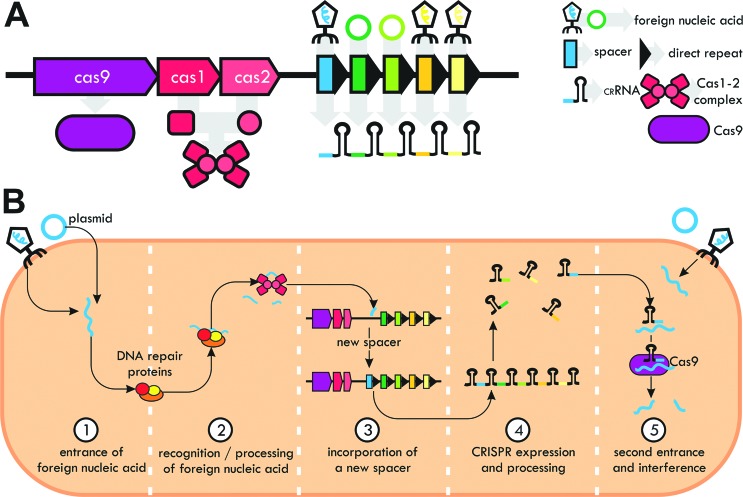
Features of CRISPR systems. (A) Main structure of a CRISPR system. For simplicity, a type II system, containing only the Cas9 protein as the interference effector, is displayed. (B) Steps of a CRISPR system activity, from foreign nucleic acid recognition and spacer incorporation (a step called adaptation) to interference.

Clustered regularly interspaced short palindromic repeats (CRISPRs), allegedly present in the genomes of close to 90% of archaea and 50% of bacteria, are a prokaryotic evolutionary defense response to the preponderance of bacteriophages in the biosphere, acting as an interference system against foreign nucleic acids (Horvath and Barrangou, 2010). The CRISPR locus is composed of direct repeats of palindromic sequences interspersed with small sequences called spacers, which are fragments of exogenous sequences (Figure 1A). A functional CRISPR system has adjacent proteins (CRISPR-associated proteins, Cas), responsible for the process of recognition of foreign nucleic acids and the interference against invasive genetic elements (Horvath and Barrangou, 2010).

CRISPR interference begins with the entrance of an exogenous DNA or RNA in the bacterial cell, which is processed into small fragments by repair proteins, like those of the RecBCD complex (Amitai and Sorek, 2016), and later recognized by a complex of universal Cas1-2 proteins, which incorporates a fragment of the invader nucleic acid in the CRISPR locus as a new spacer (Figure 1B). The CRISPR locus is expressed as a long transcript that is processed, either by Cas proteins or intrinsic RNases, into small RNAs referred to as CRISPR-RNAs (crRNAs). Each crRNA is made of a fragment of the original direct repeat and the spacer, whose complementarity to foreign mobile genetic elements is the basis of the interference process, fulfilled with the activity of another Cas protein (like Cas9), or Cas complexes (van der Oost *et al.*, 2014).

Marraffini and Sontheimer (2008) described a *S. epidermidis* CRISPR containing a spacer whose sequence was homologous to a region of the *nes* gene, coding a nickase found in virtually every staphylococcal conjugative plasmid sequenced. Consistently, this CRISPR prevented conjugation and transformation from happening, while the removal of the spacer allowed plasmid uptake. Later, Hatoum-Aslan *et al.* (2014) constructed mutants for all the nine *cas*/*csm* genes of the type III-A system in *Staphylococcus epidermidis* RP62a and showed that many mutations affected interference by impacting the interference complex formation or the crRNA biogenesis. At least 4% of the spacers within staphylococcal CRISPRs are identical to publicly available plasmid sequences, thus demonstrating their antiplasmid activity (Rossi *et al.*, 2017a). This percentage may be underrated due to under-sampling of mobile genetic elements and, as a consequence, their relative scarcity in public databases (Mojica *et al.*, 2005).

Since CRISPR-Cas systems were believed to exist in roughly 50% of bacteria, and given its antiplasmid activity, it is supposed to highly impact horizontal gene transfer among staphylococci. However, a study performed by Cao *et al.* (2016) with hundreds of *S. aureus* clinical isolates from China revealed that less than 1% of them harbored a complete arrangement of *cas* genes. Later, our group analyzed the genomes of dozens of CoNS of 15 species and also found that less than 15% of them carried CRISPRs and *cas* genes (Rossi *et al.*, 2017a). Regardless of how abundant CRISPRs really are among *Staphylococcus* isolates, they are clearly rarer than previously thought, which is consistent with the role of staphylococcal as reservoirs of antimicrobial resistance genes.

Because spacers are incorporated in the CRISPR locus following an organized and thus chronological order (van der Oost *et al.*, 2014), their sequences can be explored as molecular clocks to reveal the history of genetic invasion of a given isolate, and even to study the epidemiologic connection between different strains carrying a CRISPR of a common origin. With that prerogative, we explored the spacer sequences of CRISPRs located within SCC*mec* elements from different *Staphylococcus* species. As indicated by the high sequence identity between the SCC*mec*s, the identical sequence and organization of spacers evidenced that the mobile genetic element had been transferred between strains of canine *S. pseudintermedius* and *S. schleiferi*, and then to strains of *S. capitis* and *S. aureus* isolated from humans (Rossi *et al.*, 2017a; Rossi *et al.*, 2019).

## Concluding remarks

Most *Staphylococcus* species lack many of the *S. aureus* virulence factors and, because they are less frequently isolated from infectious processes, their impact in pathogenesis is usually overlooked. However, sequence and experimental evidences of horizontal transfer of antimicrobial resistance genes between them, favored by their capacity of forming biofilms and the scarcity of restrictive CRISPR-systems, show that these bacteria participate actively in the process of drug resistance spreading. Moreover, the fact that we exchange microbiota with our surroundings, makes it imperative that epidemiologic studies and strategies to control the advance of antimicrobial resistance consider the integrated nature of the relationship between human beings, animals, and the environment.
